# Applications of Chitosan and its Derivatives in the Treatment of Osteoarthritis

**DOI:** 10.14336/AD.2024.1080

**Published:** 2025-12-12

**Authors:** Xuexuan Fan, Guangze Chen, Sishu Wang, Xinhong Liu, Sai Huang, Cong Feng, Xiaolei Jiang

**Affiliations:** ^1^Shandong Laboratory of Biomedical Materials Engineering, Success Bio-Tech Co., Ltd., Jinan, China.; ^2^Department of Hematology, Fifth Medical Center of Chinese PLA General Hospital, Beijing, China.; ^3^National Clinical Research Center of Geriatric Diseases, Chinese PLA General Hospital, Beijing, China.; ^4^Department of Emergency, First Medical Center of Chinese PLA General Hospital, Beijing, China.

**Keywords:** Osteoarthritis, chitosan, viscosupplementation, drug carrier, scaffold for tissue engineering

## Abstract

Osteoarthritis (OA) is a common joint disease, which is mainly characterized by the degeneration of articular cartilage, inflammation of the synovial membrane of the joint, and changes in the surrounding bone tissue. With the increase of age and weight, the incidence of OA gradually increases, which seriously affects the quality of life of patients. The primary pharmacological treatments for OA include analgesics and non-steroidal anti-inflammatory drugs. However, these medications primarily provide symptomatic relief and are associated with significant side effects. Chitosan, derived from the deacetylation of chitin, has excellent biocompatibility, biodegradability, anti-inflammatory and antioxidant properties, and can promote the repair of articular cartilage. Chitosan can be used as a viscosupplement, drug carrier, cartilage tissue engineering scaffold or other forms. In this review, we systematically summarize the applications of chitosan and its derivatives in the treatment of OA.

## 1. Introduction

From a tribological perspective, human joints, particularly the weight-bearing joints of the lower limbs, operate under extremely challenging conditions. However, this cartilage undergoes aging due to increased weight-bearing and repetitive impacts over time. In humans, the aging of cartilage begins around the age of 20, as a result of body weight and external forces. While younger individuals possess a robust capacity for cartilage repair, this ability significantly declines after the age of 40, leading to noticeable degeneration of the knee joints. Consequently, some individuals begin to experience symptoms of knee OA [1].

Clinically, OA is characterized by joint pain, swelling, and stiffness, with severe cases resulting in joint deformities and functional impairments, often accompanied by secondary synovitis. The primary pathological features include progressive degeneration and destruction of articular cartilage, along with the gradual depletion of nutrients in the cartilage tissue and subsequent bone overgrowth. In the later stages, there is a reduction in chondrocytes and the formation of osteophytes. The global prevalence of OA is approximately 1%, and it tends to recur, significantly affecting the quality of life in the elderly [2].

Treatment options for OA include both non-pharmacological and pharmacological approaches. Clinically, pharmacological treatment mainly consists of analgesics (acetaminophen), symptom-relieving medications (non-steroidal anti-inflammatory drugs), and intra-articular (IA) injections (corticosteroids). However, these medications mainly provide symptomatic relief and may lead to serious adverse effects with long-term use, particularly gastrointestinal bleeding and renal impairment. Currently, there is no effective treatment specifically targeting this disease, and total joint replacement is often considered the primary therapeutic option when necessary. Similarly, joint replacement indicates some disadvantages, for example, large operative trauma and economic burden for OA patients. Thus, it is urgent to develop alternative management strategies for the patients or new treatment modes to reduce adverse reactions.

**Figure 1.**
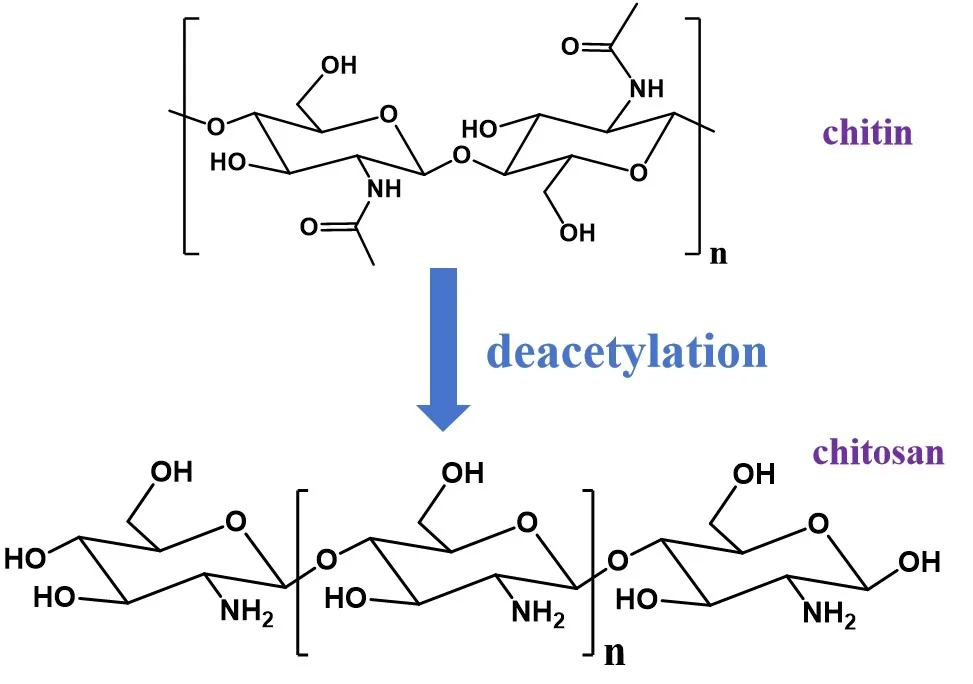
Schematic presentation of chitin deacetylation.

**Figure 2.**
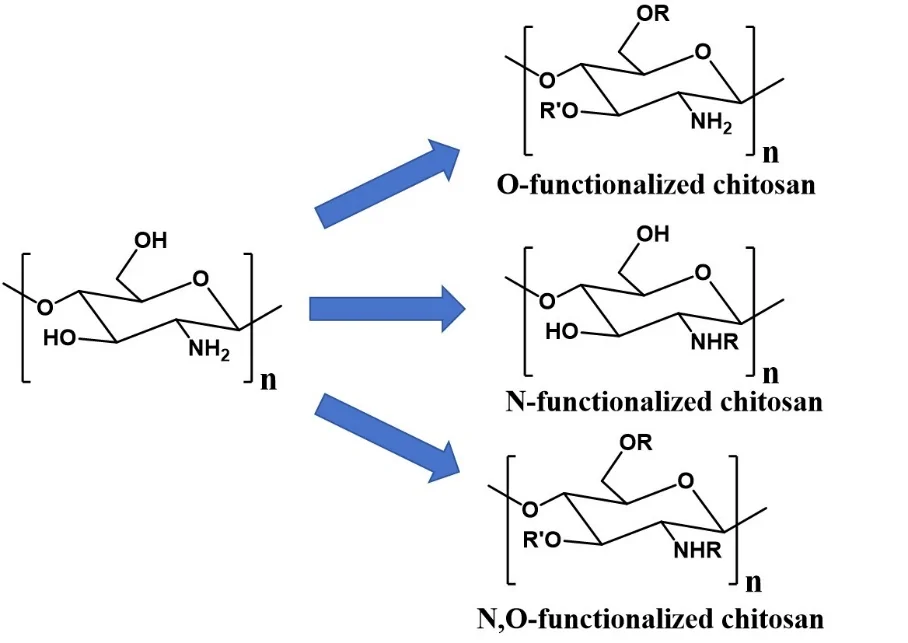
Schematic presentation of O/N/N,O-functionalized chitosan.

Chitosan, which is the deacetylated derivative of chitin (Fig. 1), has received increasing attention in recent years for its use in the treatment of OA. It is formed by the linkage of glucosamine units through β-1,4-glycosidic bonds, with a deacetylation degree typically exceeding 85%. The polysaccharide nature of chitosan, along with the presence of reactive functional groups such as hydroxyl and amino groups on the sugar units, enables it to undergo a range of chemical reactions. Hydroxyl groups can participate in alkylation, acylation, hydrogen bonding with polar atoms, and grafting reactions, while amino groups can engage in alkylation, quaternization, and reactions with aldehydes and ketones, as well as grafting. Currently, a variety of derivatives of chitosan have been synthesized targeting the O and N positions as well as the N, O positions (Fig. 2). These chemical modifications give chitosan advantages in solubility, biocompatibility, biodegradability, stability, and easy functionalization ability. All these advantageous properties have led to an expanded range of applications for chitosan in drug delivery and biomedical fields [3-9].

Chitosan can be applied in various forms for the treatment of OA, including: (i) Local injection: Direct injection of chitosan into the joint cavity as a viscosupplement to alleviate pain and inflammation locally and improve joint function. (ii) Targeted delivery system: Use chitosan-made microspheres, nanoparticles or hydrogels as drug carriers to control drug release and improve the bioavailability of drugs. (iii) Biomaterials: As a scaffold material for cartilage tissue engineering, combined with synthetic or natural materials, to prepare degradable cartilage substitutes for cartilage repair.

In this review, we systematically summarize the applications of chitosan and its derivatives as a viscosupplement, drug carrier, or cartilage tissue engineering scaffold in the treatment of OA.

## 2. Applications of chitosan and its derivatives in the treatment of viscosupplementation

Viscosupplementation therapy represents a significant breakthrough in the treatment of OA in recent years, offering a novel approach to reducing the incidence of joint diseases. This therapy involves injecting a gel-like substance into the joint space to restore the viscoelastic properties of synovial fluid, improving lubrication and cushioning in osteoarthritic joints. IA injection of hyaluronic acid (HA) is recognized as a cost-effective strategy for alleviating OA symptoms and preserving joint function. However, the short half-life of HA, typically 1-2 days in tissue, leads to a reduced retention time for HA hydrogels, thereby limiting their effectiveness. In contrast, chitosan exhibits a slower degradation rate, enhanced lubrication capacity, and superior ability to combat oxidative stress, making it a promising alternative to HA for IA applications.

Chitosan has the following characteristics, which make it an ideal candidate for Viscosupplementation: (i) Chitosan exhibits high viscoelastic properties that closely resemble those of normal synovial fluid. This allows it to mimic the physical functions of synovial fluid, enhancing joint lubrication and mitigating pressure on the articular cartilage surfaces. Moreover, chitosan can form a protective layer over the cartilage or fill gaps in degenerated cartilage, preventing harmful inflammatory cytokines present in OA synovial fluid from contacting the cartilage matrix and chondrocytes, thus safeguarding the cartilage and reducing the risk of joint adhesions and degeneration. (ii) Unlike the metabolic degradation of HA, chitosan is primarily eliminated through cellular phagocytosis, a process that has been shown not to induce significant cytokine-mediated inflammatory responses locally. (iii) The final degradation product of chitosan is glucosamine, which is a natural component of cartilage tissue, has outstanding biocompatibility and does not invoke adverse foreign body reactions. The glucosamine monomers can significantly contribute to the synthesis of proteoglycans, vital constituents of the cartilage matrix, thus replenishing lost proteoglycan levels. (iv) Chitosan can inhibit the synthesis of Matrix Metalloproteinases and the nitric oxide production, thereby reducing the synthesis and release of inflammatory mediators and reducing synovial inflammation [10-13].

**Figure 3.**
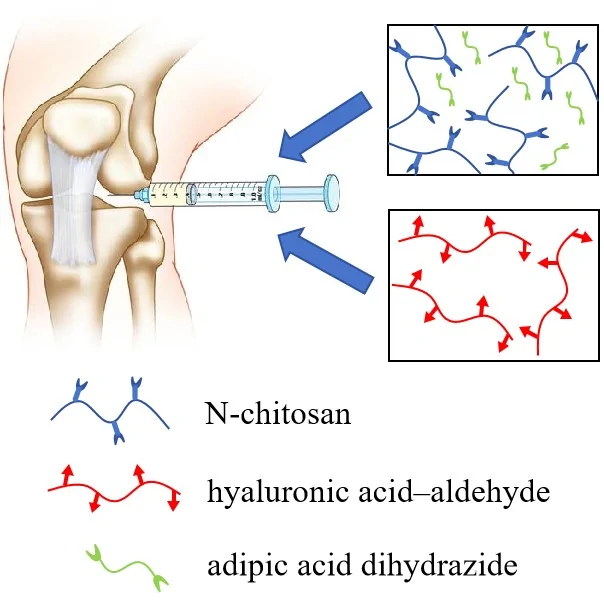
Chitosan-based hydrogel is prepared by *in situ* crosslinking of N-chitosan, adipic acid dihydrazide, and hyaluronic acid-aldehyde, and injected into the articular cavity [14].

As shown in Figure 3, Mou et al. developed an injectable, self-healing hydrogel for IA injection therapy in OA through the *in situ* crosslinking of N-chitosan and adipic acid dihydrazide with hyaluronic acid-aldehyde [14]. This supramolecular hydrogel demonstrates excellent biocompatibility with chondrocytes. IA administration of this novel hydrogel significantly reduces the inflammatory microenvironment in knee joints by inhibiting the release of inflammatory cytokines in the synovial fluid and cartilage, including TNF-α, IL-1β, IL-6, and IL-17. Histological and behavioral assessments indicate that this chitosan-based supramolecular hydrogel not only effectively regulates the inflammatory microenvironment but also retards cartilage degeneration and alleviates pain in OA rat models.

## 3. Applications of chitosan and its derivatives in drug carriers

Controlled and targeted drug delivery systems in the field of biomedicine represent a significant area of research. Direct delivery of drugs to the lesion site by a specific delivery mode can increase the drug concentration at the target site, enhance the therapeutic effect, and reduce systemic side effects.

Chitosan can be used as a drug carrier for the treatment of OA due to its good biocompatibility, biodegradability and anti-inflammatory properties. As a drug carrier, chitosan can effectively carry a variety of drugs, including growth factors, non-steroidal anti-inflammatory drugs, glucocorticoids, antibodies and joint protective agents. Chitosan can provide protection for the drug, reducing its degradation and inactivation in vivo. This is particularly important for drugs that can be easily metabolized, such as certain proteins and biologic drugs, thereby improving their bioavailability. The drug-loading mechanism of chitosan mainly includes: (i) Chitosan is protonated under acidic conditions and is capable of electrostatic attractive interactions with anionic drugs; (ii) Chitosan molecules contain active groups such as amino groups and hydroxyl groups, which can form hydrogen bonds with hydrogen bond donors or acceptors present in drug molecules. (iii) The porous structure of chitosan enables physical adsorption of drugs. On the one hand, by surface modification of chitosan (binding to targeted molecules related to arthritis such as collagen and glucosamine), its aggregation to diseased tissues can be enhanced. On the other hand, according to the characteristics of the internal joint environment, the hydrophilicity of chitosan can be appropriately adjusted to improve the drug distribution in the joint fluid. This targeting helps to reduce the side effects of drugs at non-target sites and improve the therapeutic effect. The degree of cross-linking and preparation parameters of chitosan are adjustable, which can be designed to achieve controlled drug release, prolong the duration of drug effect, and reduce the frequency of administration, which is very important for relieving the symptoms of OA [15]. Furthermore, its ease of modification and moldability offers versatility for multifunctional applications, enabling the fabrication of various chitosan-based materials, such as microspheres, nanoparticles, and hydrogels, which can serve as drug carriers. The differences between these three vectors in the treatment of OA are discussed in detail below.

### 3.1. Chitosan microspheres

Chitosan microspheres are small particles composed of chitosan, with preparation methods including spray drying, emulsion polymerization, and phase separation. The size of chitosan microspheres ranges from 1 micron to 1000 microns, exhibiting a relatively uniform shape that allows for the control of surface area and porosity. Drugs can be loaded into these microspheres through physical entrapment or chemical crosslinking, enabling prolonged drug release and reducing the frequency of administration. Additionally, the surface of the microspheres can be modified with specific antibodies or ligands to enhance targeting to diseased tissues, thereby increasing the concentration of the drug at the joint site and improving its bioavailability [16-18].

For example, Zhang et al. observed an increased expression of Yes-associated protein in the chondrocytes of patients with OA [19]. Treatment of chondrocytes with the selective Yes-associated protein inhibitor verteporfin effectively suppressed OA progression. To achieve a more effective sustained release of verteporfin, they designed a chitosan microsphere system. This system facilitates an initial rapid release of the drug, followed by a gradual release, ensuring that the drug exerts its effects over the required time frame. In a mouse model of OA, animals treated with the chitosan microsphere verteporfin sustained-release system exhibited significantly less cartilage loss and damage compared to control animals, demonstrating the in vivo applicability of the chitosan microsphere system.

### 3.2. Chitosan nanoparticles

Chitosan nanoparticles are nano-sized particles composed of chitosan, with preparation methods including self-assembly, solvent evaporation, ultrasonic techniques, and co-precipitation. The size of chitosan nanoparticles typically ranges from 1 nanometer to 100 nanometers, exhibiting a large specific surface area. By adjusting the preparation conditions, it is possible to modify their particle size, surface charge, and morphology, optimizing drug release rates and enhancing biological activity. Nanoparticles of this size can be more effectively internalized through cellular endocytosis, facilitating their ability to cross biological barriers and improving drug bioavailability. Surface modifications, such as PEGylation or conjugation with antibodies, can enhance cellular uptake at specific targets, enabling targeted delivery. This allows chitosan nanoparticles to act directly on the joint surface or specific cell populations, such as chondrocytes [20,21].

For example, Kartogenin was recently reported as a disease-modifying OA drug that promotes cartilage repair, but its therapeutic effect is impeded by its very low solubility. By wet milling and spray drying, Luca et al. designed a nanocrystal-chitosan particle intra articular delivery system for OA treatment. The kartogenin nanocrystals increased the solubility and dissolution rates of kartogenin [22].

### 3.3. Chitosan hydrogels

Chitosan hydrogels are three-dimensional crosslinked polymer networks composed of chitosan, with preparation methods including physical crosslinking, chemical crosslinking, solvent evaporation, and self-assembly. These hydrogels leverage the favorable properties of chitosan, exhibiting excellent biocompatibility and biodegradability. Due to their ability to absorb large amounts of water, chitosan hydrogels structurally resemble natural cartilage, providing enhanced flexibility and biomimetic characteristics. This property allows them to maintain a hydrated state within the joint cavity for extended periods, facilitating lubrication. The drug release rate from the hydrogels can be modulated by adjusting factors such as pH, temperature, crosslinking density, network structure, and drug loading capacity. Furthermore, the pore size of the hydrogel and the hydrodynamic characteristics of the drug are important attributes that govern the diffusion efficiency of the encapsulated drug. Chitosan hydrogels have garnered significant attention from researchers as drug delivery systems [23,24].

For example, Chen’s team dispersed chitosan microspheres prepared by using a spray-drying method in a thermally responsive chitosan hydrogel. These microspheres incorporated into the hydrogel were loaded with anti-inflammatory drugs and injected into the knee joints of OA rabbits. The results showed that the composite hydrogel released the drugs for more than 7 days in a controlled manner [25].

**Table 1:** Differences among the C hitosan microspheres, nanoparticles and hydrogels.

	Structure	Advantages
**Chitosan microspheres**	1-1000 μm, porosity	drug embedding, sustained release
**Chitosan nanoparticles**	1-100 nm, large specific surface area	endocytosis, high bioavailability
**Chitosan hydrogels**	3D network, water swelling	biomimetic, lubricous

Chitosan microspheres, chitosan nanoparticles, and chitosan hydrogels each possess unique drug carrier characteristics that cater to different therapeutic needs in the treatment of OA (Table 1). Chitosan microspheres are particularly suited for drugs requiring extended release, offering improved targeting and controlled release capabilities. Chitosan nanoparticles are advantageous for enhancing drug bioavailability and internal cellular uptake, especially for delivering certain biopharmaceuticals. Chitosan hydrogels are ideal for applications that necessitate sustained lubrication and drug release within the joint, as they can maintain a hydrated state while providing favorable drug release properties. When selecting an appropriate drug carrier, a comprehensive assessment based on specific therapeutic requirements and drug characteristics is essential.

## 4. Applications of chitosan and its derivatives in cartilage tissue engineering

**Figure 4.**
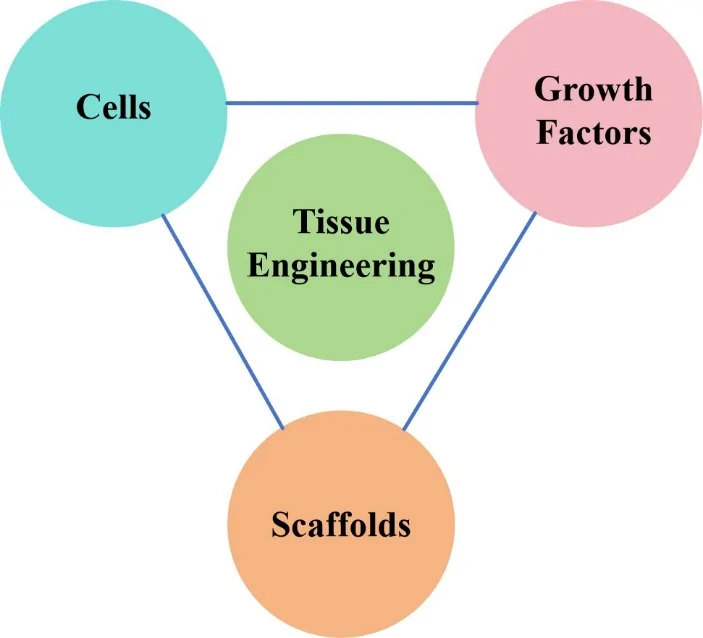
The elements of tissue engineering.

The onset of OA is typically associated with cartilage damage and degeneration, leading to an imbalance in cartilage metabolism, chondrocyte apoptosis, and matrix degradation. Due to the avascular, aneural, and anastomotic characteristics of articular cartilage, self-healing after injury is challenging. While IA injections and targeted drug delivery primarily help alleviate inflammation and pain, they do not repair the damaged joint. Cartilage tissue engineering involves the combination of biomaterials, cells, and growth factors to promote the regeneration of defective cartilage. This regenerative medicine approach often utilizes therapies such as stem cells or recombinant proteins to facilitate cartilage regeneration and improve both the function and structure of the joint (Fig. 4) [26-28].

Chitosan is utilized in the fabrication of scaffolds for cartilage tissue engineering due to its excellent biocompatibility and biodegradability. The porosity and pore size of chitosan scaffolds can be tailored to optimize cell infiltration and nutrient transport. In vitro cultured chondrocytes can attach, proliferate, and differentiate on chitosan scaffolds. Furthermore, chitosan can serve as a carrier for bioactive molecules such as growth factors and cytokines, which further enhance the synthesis of cartilage matrix components, including collagen and proteoglycans by chondrocytes [29-35].

For example, Shen et al. introduced poly (lactic-co-glycolic acid) short fibers and cartilage-decellularized matrix into chitosan-based hydrogels to develop cartilage tissue engineering scaffolds with highly biomimetic characteristics [36]. The incorporation of poly (lactic-co-glycolic acid) short fibers structurally enhances the biomimetic properties of the chitosan-based hydrogels, providing physical cues that promote cell growth and cartilage differentiation while fulfilling the mechanical support requirements needed after scaffold implantation. Meanwhile, cartilage-decellularized matrix, obtained through the decellularization of natural cartilage tissue, retains a wealth of native cartilage bioactive components and exhibits excellent cartilage-inductive activity as well as in situ cell recruitment capabilities, significantly improving the effectiveness of cartilage repair and regeneration. This study demonstrates the substantial potential of the developed highly biomimetic chitosan-based cartilage scaffolds for the repair and regeneration of articular cartilage damage, while also providing a solid foundation and possibility for the clinical translation of chitosan-based biomaterial scaffolds in joint cartilage repair.

## 5. Conclusion

OA is a prevalent condition among middle-aged and elderly individuals, leading to severe joint pain and deformities that significantly impair their quality of life. As the aging population continues to grow, this issue has become increasingly pronounced, drawing heightened attention to potential solutions. Chitosan, derived from chitin, exhibits excellent biocompatibility, biodegradability, and bioactivity, garnering increasing attention for its applications in the treatment of OA in recent years. Chitosan can be applied in various forms for the management of OA, including: (i) IA injection: Direct injection of chitosan into the joint cavity alleviates pain and inflammation through local action, thereby enhancing joint function. (ii) Targeted delivery systems: Chitosan-based microspheres or nanoparticles serve as drug carriers, allowing for controlled drug release and improved bioavailability. (iii) Biomaterials: As scaffold materials, chitosan can be combined with other synthetic or natural materials to create biodegradable cartilage substitutes for cartilage repair. In the treatment of OA, IA injection, targeted drug delivery, and cartilage tissue engineering each offer unique advantages and applications: IA injection can restore joint lubrication and reduce friction, making it suitable for short-term pain relief; targeted drug delivery enhances therapeutic effects with minimal systemic side effects, while cartilage tissue engineering provides a long-term solution for patients seeking to improve cartilage damage and joint function. As researchers continue to explore and develop, the role of chitosan in OA treatment is poised to expand, offering new possibilities for future therapeutic strategies.
